# The development of a global research agenda and individual participant data platform for visceral leishmaniasis: challenges and future opportunities

**DOI:** 10.1186/s13071-025-07121-2

**Published:** 2025-12-02

**Authors:** Sauman Singh-Phulgenda, Prabin Dahal, Brittany J. Maguire, Jorge Alvar, Fabiana Alves, Mitali Chatterjee, Carlos Costa, Simon L. Croft, Philippe J. Guerin, Dinesh Mondal, Ahmed Musa, Krishna Pandey, Koert Ritmeijer, Gustavo Romero, Shyam Sundar

**Affiliations:** 1 Infectious Diseases Data Observatory (IDDO), Oxford, UK; 2 Centre for Tropical Medicine and Global Health, Nuffield Department of Clinical Medicine, University of Oxford, Oxford, UK; 3 Royal Academy of Medicine of Spain, Madrid, Spain; 4 Drugs for Neglected Diseases Initiative, CH1202 Geneva, Switzerland; 5 Institute of Postgraduate Medical Education & Research (IPGMER), Kolkata, India; 6 Federal University of Piauí, Teresina, Piauí Brazil; 7 Faculty of Infectious and Tropical Diseases, London School of Hygiene and Tropical Medicine, London, UK; 8 International Centre for Diarrhoeal Disease Research, Dhaka, Bangladesh; 9 Institute of Endemic Diseases, University of Khartoum, Khartoum, Sudan; 10 ICMR–Rajendra Memorial Research Institute of Medical Sciences (RMRIMS), Patna, India; 11 Médecins Sans Frontières, Amsterdam, The Netherlands; 12 Center for Tropical Medicine, University of Brasilia, Brasilia, Brazil; 13 Infectious Disease Research Laboratory, Department of Medicine, Institute of Medical Sciences, Banaras Hindu University, Varanasi, India

**Keywords:** Kala-Azar, Collaboration, Platform, Research agenda, Data Re-use

## Abstract

**Background:**

Visceral leishmaniasis (VL) is one of the neglected tropical diseases (NTDs) listed by the World Health Organization (WHO). The disease is currently in the elimination phase in the Indian subcontinent (ISC) and being targeted for elimination by 2030 in East Africa (EA). Maintaining the necessary financial and political commitments to achieve and sustain the current elimination efforts remains challenging. As with other NTDs, VL research is constrained by limited funding, and drug development has relied largely on partnerships between not-for-profit organisations and the pharmaceutical industry. Conducting robust clinical studies remains difficult, and therapeutic innovations have been limited. However, re-use of existing data offers an untapped opportunity to generate new evidence.

**Methods:**

We describe the process of developing a global VL research agenda and the establishment of an individual participant data (IPD) platform at the Infectious Diseases Data Observatory (IDDO). Key steps included a systematic scoping review of VL clinical trials, consultations with the Scientific Advisory Committee, expert and public reviews, and implementation of an equitable governance framework to harmonise and share IPD.

**Results:**

The VL research agenda, finalised in 2019, identified priority methodological and clinical questions suited to IPD analyses. The IDDO VL platform currently hosts harmonised data from nearly 15,000 patients across more than 50 studies (VL and post-kala-azar dermal leishmaniasis, PKDL). The platform is an inclusive resource guided by an equitable governance framework and provides a critical asset to support new evidence generation and can serve as a historical data to support accelerated drug development.

**Conclusions:**

The development of a global VL research agenda has provided an inventory of priority research questions of public health importance. A shared IPD platform aligned with this agenda was developed to complement ongoing global efforts. In addition, such a platform can accelerate secondary evidence generation, support methodological innovation and inform future trial designs and policy. Sustained collaboration and investment are needed to maximise the scientific and public health value of data re-use in VL and PKDL.

**Supplementary Information:**

The online version contains supplementary material available at 10.1186/s13071-025-07121-2.

## Background

The Infectious Diseases Data Observatory (IDDO) is a data platform launched in 2016 to collate and harmonise individual participant data (IPD) from studies on infectious diseases [1]. This was built upon the success and experience of the WorldWide Antimalarial Resistance Network (WWARN), the prototypic model of IDDO. The underlying ethos of the IDDO platform has been to promote collaborative science by enabling researchers to address questions of public health importance from existing datasets, and promote data re-use, thus optimising scientific utility. Prime examples include facilitating large-scale individual participant data meta-analysis (IPD-MA) that generated evidence to support the World Health Organization (WHO) recommendations for paediatric dose-optimisation for one of the frontline antimalarial drugs [2, 3] and for treatment of malaria in pregnancy [4]. IDDO has created a transparent data governance framework to realise this and built trust within the scientific community through an equitable data ownership model. In particular, IDDO promotes a model where investigators who generated the primary study remain the data controller and are engaged in subsequent scientific projects when their shared data are re-used [5]. This data governance framework has been the subject of a scholarly evaluation and is well-described in literature [6].

Over the past decade, IDDO has engaged with a number of research communities to map the landscape of clinical trials for several diseases beyond malaria, and, in particular, for diseases identified by the WHO as neglected tropical diseases (NTDs) [7–12]. NTDs disproportionately affect women and children in impoverished communities from low- and middle-income countries (LMICs) and nearly a billion people globally. Visceral leishmaniasis (VL) is one of these and remains endemic in countries from the Indian subcontinent (ISC), East Africa (mainly Sudan, South Sudan, Ethiopia, Uganda and Kenya), South America (predominantly Brazil) and the Mediterranean region [13]. Over the past decade, there has been a progressive decline in the global VL burden, the decline being attributed to the strong political commitment in the ISC region that led to the establishment of the Kala-Azar Elimination Programme (KAEP) in 2005 [14]. Bangladesh achieved a historic milestone in 2023, becoming the first country to be validated for the elimination of VL as a public health problem [15]. India is currently on the brink of achieving the targets, with low case numbers reported in 2024 [16]. Similarly, low case numbers have been reported in Nepal, but six districts are still considered endemic [17]. The success model of the elimination programme in the ISC has inspired a similar initiative recently launched in East Africa [18]. While the burden of VL has progressively declined in the ISC region, new challenges are emerging, such as treatment of VL relapses and safety concerns regarding treatment of post-kala-azar dermal leishmaniasis (PKDL) with miltefosine [19, 20], both of which require urgent attention.

Generating robust, up-to-date evidence remains paramount to not only preserve the epidemiological gains in the ISC but also facilitate a successful elimination campaign in East Africa. In particular, there is limited knowledge on the optimal treatment approaches and package of care from patient groups frequently excluded from therapeutic efficacy studies, such as pregnant women [21], patients with co-infections such as tuberculosis or severe co-morbidities, very young patients [22] and patients with PKDL [12]. The efficacy of anti-leishmanial therapies remains regional [23], and the transportability of findings from studies conducted in one region is largely considered inappropriate owing to the regional heterogeneity, which warrants caution.

The IDDO VL data platform, which currently hosts standardised datasets on nearly 15,000 patients in VL and PKDL (2000–2021), can contribute to addressing some of these challenges [24]. We summarise some of the key steps involved in the evolution of the VL platform, including the development of a research agenda and highlight research generated by the platform to date, and discuss the future potential of this platform.

## Methods

### Key underlying steps and evolution of the IDDO VL data platform

#### Feasibility assessment of IPD platform development

The development of the VL data platform followed a multi-phase process, and here we detail pivotal steps, including the feasibility assessment and engagement of stakeholders. An important pillar of the first phase of platform development, also referred to as the scoping phase, was to conduct a scoping review of the scientific literature. This was undertaken with the overall aim of identifying the existing volume of published VL clinical trial data, along with evaluating the technical feasibility and clinical value of collating and standardising IPD [8]. The initial review identified 145 prospective clinical trials published between 1980 and 2016 with 26,986 patients treated with anti-leishmanial therapy. A substantial proportion of the studies identified being published on or after 2000. These studies also provided a comprehensive source of the individual researchers and institutions involved in VL research globally. Initial engagement with key stakeholders during the scoping phase was essential to ensure any platform established was supported and would be equitable and adapted to meet the specific research requirements of the VL community. Overall, the scoping phase concluded that the need existed, and given the sufficiently large volume of studies and enrolled patients, development of a data platform was feasible and would add value to the global VL research community. The initial scoping review has now evolved into a living mapping review, which is periodically updated with the review database available as an open public resource (VL clinical trials library, International Prospective Register of Systematic Reviews [PROSPERO]: CRD42021284622).

#### Iterative development of a research agenda

**Fig. 1:**
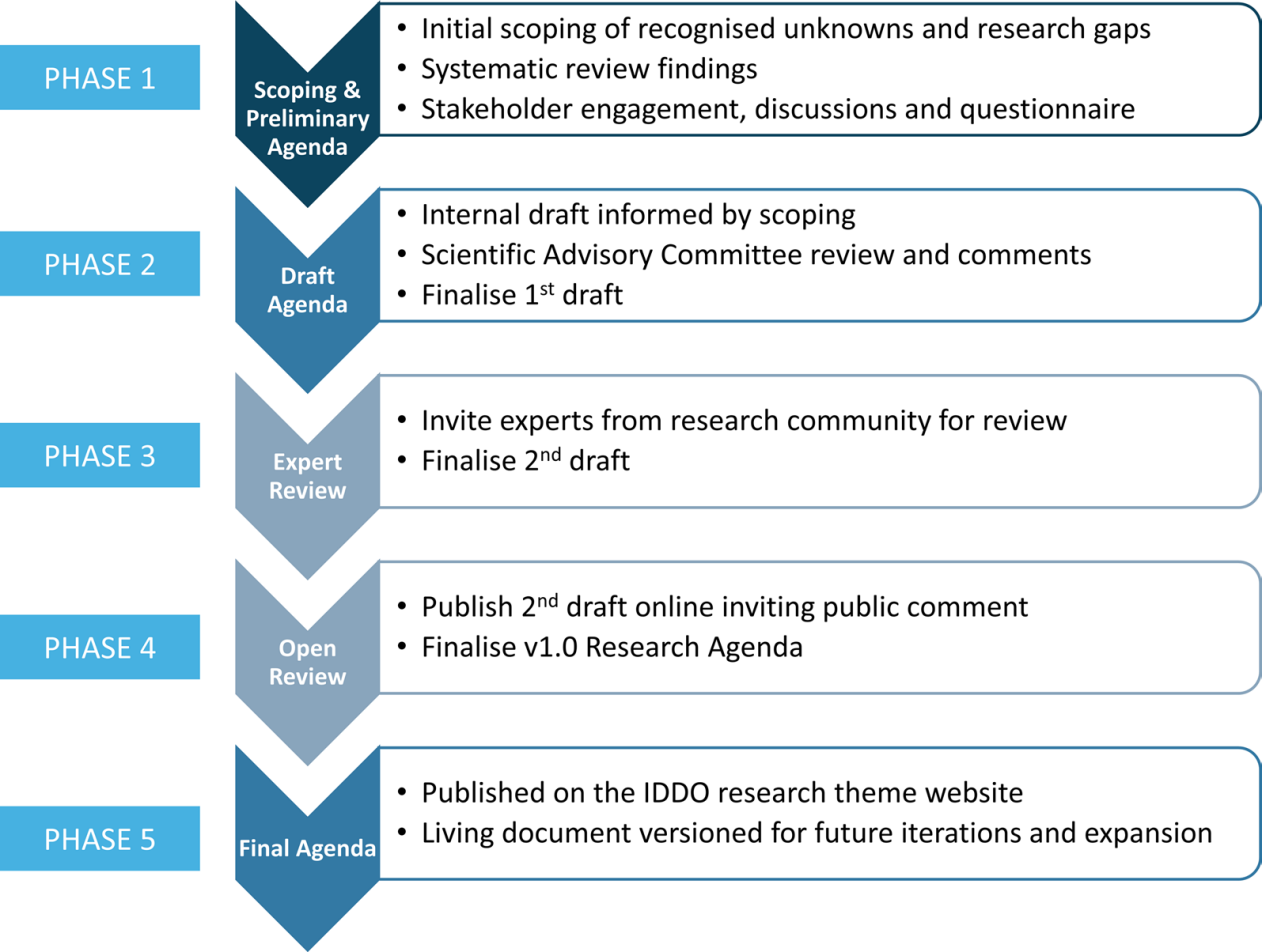
IDDO research agenda development

Following the conclusion of the feasibility assessment, a research agenda was then developed in consultation with key VL stakeholders (Fig. 1). This process was partly informed by a previous research and development agenda proposed in 2002 [25]. Since the initial proposal in 2002, epidemiology of the disease and the available anti-leishmanial armamentarium have changed dramatically, thus providing a rationale for an updated agenda that reflected the current needs of the global community.

The research agenda aimed to identify the key scientific questions that could be addressed using a standardised database of individual-level data from patients enrolled in clinical trials. The research agenda development was an iterative process, with the document to be reviewed by the IDDO VL Scientific Advisory Committee (SAC) on a periodic basis. The first draft of the research agenda was developed in 2018, facilitated by the IDDO secretariat, which developed a tiered approach consisting of four phases (Fig. 1). The first stage was to revise the 2002 agenda and identify current research gaps via a scoping review of literature. At the second stage, the IDDO VL SAC created a first draft of the research agenda. The third stage was disseminating the draft agenda to key VL experts identified by the SAC members. The experts had two months to provide their comments, which were then collated and synthesised to produce a second version of the draft agenda. The final step of this inclusive process was opening the research agenda for wider public consultation. The latest version was published on the IDDO website, and an invitation was disseminated for additional public comments or feedback over a 2-month period (May–June 2019) [26]. The systematic scoping review of clinical trials identified the target audience for this public consultation and was further shared with the attendees of “International Conference on Innovations for the Elimination and Control of Visceral Leishmaniasis (IEC-VL)” (New Delhi, India, 28–30 November 2018). Overall, the research agenda was designed to encourage collaboration and involvement of the leishmaniasis scientific community to identify priority research areas. Furthermore, it was considered that the research agenda would act as a tool to make science the driver of facilitating data re-use and maximising the scientific utility of valuable data. The current version of the research agenda was finalised in June 2019 and is available on IDDO’s webpage [26].

#### Development of the individual participant data platform

Alongside engagement with the VL community in developing a research agenda, IDDO began actively soliciting datasets from clinical trials identified in the initial scoping review. In a pilot phase of development of the platform, the IDDO VL SAC, which comprised experts who had undertaken several of these clinical trials, shared data from their studies [27]. This facilitated characterising heterogeneity in standards adopted across the trials and across the region. A common data standard was then adopted that was compliant with the Clinical Data Interchange Standards Consortium (CDISC) format. By the end of 2019, datasets from 29 studies were uploaded to the IDDO VL platform (6418 patients) [24], with a critical mass of datasets reached to enable undertaking IPD-MA.

On the basis of the questions identified in the research agenda, the SAC endorsed two research questions as a priority. This included undertaking two IPD meta-analyses: (i) identification of host, parasite and drug determinants of treatment failure and (ii) assessment of haematological safety following treatment initiation. The authors of the relevant studies identified through the scoping review (*n* = 147 eligible publications) were invited to collaboratively participate in the development of the VL data platform and contribute to the two proposed IPD-MAs in May 2021. IPD from some of the eligible studies had already been part of the VL data platform (through the SAC), and overall, there were 4 submissions made in 2018, 25 in 2019, 14 in 2020, 12 in 2021, and 3 in 2022 [24]. Outside of the data submitted for the purpose of the two proposed IPD-MAs, the platform also currently holds studies on PKDL and further VL data generated from observational research. Currently, data from more than 50 different submissions describing IPD from nearly 15,000 patients (VL, PKDL included) are held in the platform.

A key challenge in the development of the data platform has been the non-retrieval of data from a large number of studies, in particular from the studies conducted prior to 2000 [28]. Of the studies indexed in the IDDO VL trials library, IPD was retrievable from approximately one-fifth of them. In some studies, even when the study investigators were interested in sharing the IPD, the cost associated with the digitisation of historical paper records made it unfeasible to retrieve these datasets in a usable electronic format. When data was submitted to the IDDO data platform, limited funding was available to support the data curation, and delays in response to data-related queries from the primary investigators posed logistical challenges. Further details regarding the retrieval of requested data and underlying challenges are discussed elsewhere [28].

#### Governance framework of the data platform

IDDO has developed an inclusive and equitable data governance framework that allows primary investigators to remain the data controller (Box  1). IDDO’s model supports the engagement of data controllers in subsequent scientific projects when their shared data are re-used [5]. When an investigator submits primary data to the platform, they can choose the right to control the data either through (i) a data access committee (DAC), which is independent of IDDO and comprises members of the infectious disease scientific community, or (ii) retain the decision of data-sharing rights. The DAC is currently chaired by the WHO/TDR (the Special Programme for Research and Training in Tropical Diseases) [29]. The DAC reviews the proposed IPD-MA and decides on access to re-use the data on the basis of clarity of the research question and methodology. In addition, a key necessity is that the data requester has to sign a data transfer agreement to safeguard the patients’ privacy. If the data contributors keep the decision of data-sharing rights with them, this means that every time a request is made to re-use the data from the study the investigator will review the research proposal and decide on whether to allow their data to be re-used or delegate this task to the DAC [5].

**Box 1:** Terminology related to the IDDO’s governance framework and IDDO’s open-access resources

Terminology	Description
VL clinical trials library	An open-access library of published VL clinical studies, maintained periodically by the IDDO team. The library is based on a systematic search of literature with an open and transparent search protocol [100] PROSPERO registration: CRD42021284622
PKDL clinical trials library	An open-access library of published PKDL studies. The library is based on a systematic search of literature with an open and transparent search protocol [12]. As opposed to the VL clinical trials library, the PKDL trials library indexes not only trials but also observational research such as case-series PROSPERO registration: CRD42021295848
IDDO VL surveyor	A website displaying all the VL and PKDL trials identified in the IDDO VL and PKDL clinical trial libraries. URL: https://www.iddo.org/tool/vl-surveyor
IDDO VL data platform	Individual participant data repository of published (and unpublished) VL and PKDL studies standardised by IDDO to a Clinical Data Interchange Standards Consortium (CDISC)-compliant common storage format. The database remains open to the scientific community who can request access through the IDDO’s data access system which is controlled by a data access committee (DAC). URL: https://www.iddo.org/vl/data-reuse/accessing-data
IDDO VL scientific advisory committee (SAC)	The SAC advises on all technical and research activities for the IDDO VL data platform. Appointed from across the VL and broader scientific research community, the SAC members have significant expertise in research, policy and funding acquisition. URL: https://www.iddo.org/vl/governance/scientific-advisory-committee
IDDO data access committee (DAC)	The DAC is independent to IDDO and is currently overseen by TDR, the Special Programme for Research and Training in Tropical Diseases, on behalf of World Health Organization (WHO). Decisions are made on behalf of the data contributors who have instructed that this responsibility be delegated to the DAC, and access is managed according to the Data Access Guidelines. The DAC reviews the scientific merit and importance of the proposals requesting the re-use of the data in the IDDO platform. URL: https://www.iddo.org/governance/malaria-ntd-data-access-committee

All the URLs were accessed on 30 September 2025

## Results

### Questions identified in the research agenda and the feasibility of addressing them

#### Methodological questions

The research agenda identified 12 broad methodological questions (Additional File 1). This included characterisation of the variation in study design, conduct, analysis and reporting, as well as understanding the impact of such variations in terms of reported efficacy and possible generalisation of results beyond the study population. A key question identified was regarding the impact of follow-up duration on the derived efficacy estimates. The majority of VL clinical trials have a patient follow-up duration of 6 months, with some studies extending to 12 months. The question regarding the optimal follow-up duration to capture late relapse is of public health importance in terms of disease control and elimination since relapse provides an infective pool of parasites that can sustain disease transmission and, thus, remains crucial to achieve VL elimination [30].

Some of the methodological questions have been partly addressed through systematic literature reviews using IDDO’s open-access VL clinical trials library [22, 31]. For example, the characterisation of inclusion/exclusion criteria adopted and different aspects of trial design, conduct and analysis have been recently summarised [22]. Similarly, using the same library, an aggregate data meta-analysis of 21 efficacy studies with a follow-up duration longer than 6 months was undertaken and concluded that an estimated quarter of all observed relapses were reported after 6 months of follow-up [31]. This observation has also been reported in a retrospective analysis of data from routine care settings in South Sudan, where it was found that over 15% of all relapses occurred after 1 year [32]. However, weighing the benefits of extended follow-up versus the associated increase in financial and logistical challenges requires serious consideration.

The IDDO VL data platform provides further opportunity to explore the optimal follow-up duration by taking exact relapse time and patient-level covariates into consideration. In addition, the data platform can permit methodological development by facilitating comparison of different analytical approaches for estimating efficacy and handling loss to follow-up and missing data.

#### Clinical features, host, parasite and drug determinants of drug efficacy

The research agenda identified 15 questions regarding clinical features and therapeutic outcomes (Additional File 1). It has been well-established that the efficacy of drug regimens to treat VL is highly regional. Single-dose liposomal amphotericin B (L-AmB) at a 10 mg/kg dosage is currently used as the first-line therapy in the ISC but is sub-optimal in East Africa [23, 33]. While pentavalent antimony is no longer the main drug in ISC owing to resistance, a combination regimen of pentavalent antimony and paromomycin remains in use in East Africa and has demonstrated high efficacy [23, 33]. The variation in treatment response observed across regions is likely due to multiple factors. These include regional parasite diversity, differences in the pharmacokinetic and pharmacodynamic properties of drugs across patient populations and variations in patient characteristics, particularly host immunity [34–37]. Many studies have explored the determinants of treatment outcomes across different geographical regions and patient populations [38–43]. However, a reliable assessment of such risk factors requires a large number of events. As the number of initial failures and relapses observed in any single study is often small [31], it is extremely difficult to assess determinants of treatment outcome with precision in a single study, thus necessitating an IPD-MA approach.

The IDDO VL data platform is currently facilitating two IPD-MAs aimed at characterising treatment outcomes. The first one explicitly aims to assess the risk factors for different therapeutic outcomes across different regions [44]. The second IPD-MA focuses on the development of a clinical algorithm for estimating the risk of relapse following treatment, with the aim of being used as an easy bedside tool in field conditions [45]. This work is currently ongoing and can be a useful tool for optimising available resources and ensuring efficient patient management [46].

#### Separation of treatment failure from drug resistance

An important distinction that needs to be made is between drug susceptibility, drug sensitivity and drug resistance, as well as the separation of treatment failure from drug resistance [47]. Drug resistance arises as a result of selection under drug pressure, and the genetic changes/mutations that lessen the parasite’s susceptibility can be heritable [37, 48]. Several factors can facilitate the evolution of drug resistance, such as underdosing, the use of sub-standard medicines or drug misuse, including poor patient adherence. For example, a drug with a short half-life and high therapeutic ratio (such as amphotericin B) has a lower probability of selecting resistance, whereas a long half-life and a low therapeutic ratio (e.g. miltefosine) leaves the parasites exposed to sub-therapeutic levels and provides a scenario conducive for the emergence of resistance [49]. Underdosing has been a major problem with miltefosine [50–52], especially in children who are usually at a greater risk of being under-exposed, as the drug clearance is faster. Hence, allometric scaling was proposed to improve drug exposure in paediatric populations for this regimen [53] with improved treatment response [54, 55]. The IDDO VL data platform provides further opportunity to explore the dose–response relationship for anti-leishmanial therapies through the proposed IPD-MA [44].

#### Post-kala-azar dermal leishmaniasis (PKDL)

PKDL, a skin rash presenting as a sequelae of VL, occurs in approximately 3–10% of patients in the ISC within 4 years vis-a-vis 50–60% in Sudan within 6 months of antimony treatment [56]. More recent data, however, show 21% of patients with VL developing PKDL following Sodium Stibogluconate (SSG)-Paromomycin (PM)  therapy [55], although the exact case burden remains unclear [57]. Patients with PKDL can act as a reservoir of *Leishmania* parasites, particularly in the inter-epidemic period; the West Bengal VL outbreak in the 1980s is widely thought to have been initiated by PKDL cases [58]. Xenodiagnosis studies have confirmed that patients with PKDL carry *Leishmania* parasites and may serve as an infective source for phlebotomine sand flies [30, 59, 60]. Prompt and optimal case management of PKDL is therefore essential to reduce this parasite reservoir [57, 61].

Overall, the research agenda identified eight PKDL-related research questions (Additional File 1). In particular, the longer healing time, especially for macular PKDL cases in South Asia, and the need for a prolonged PKDL treatment compared with VL, exposes patients with limited skin symptoms to higher toxicity risks and adds to the costs pertaining to the programme. Various treatment regimens adopted in the ISC in the past included sodium stibogluconate 20 mg/kg for 120 days, extendable to 200 days in case of unsatisfactory response [62]. More recently AmBisome (15 mg/kg) over 15 days, a 12-week course of miltefosine, and a combination of AmBisome and miltefosine have been tested [63]. In Sudan, treatment recommendations include a lengthy 60–90 days of antimonial therapy or L-AmB 50 mg/kg total dose. Young children (< 9 years) are known to be at an increased risk of development of PKDL (compared with ≥ 45 years) in Sudan [64], whereas in India, age < 12 years and female patients have an increased risk of developing PKDL [65]. However, a clear understanding of the risk factors for failure to PKDL treatment across different settings is lacking, as the majority of the PKDL studies are relatively small to investigate the putative factors of failure [12].

In addition, identification of risk factors for PKDL development following VL treatment requires a long-term follow-up of patients, such as the 4-year cohort study from Bangladesh, which characterised the incidence of PKDL by drug regimens [66], and the study from India, which followed up patients for at least 2 years [65]. Such longitudinal studies can help in identifying patient factors that enable early profiling of patients at a high risk of subsequent PKDL. Such studies are particularly relevant as there is no vaccine for VL or PKDL, and this is unlikely for at least the foreseeable future [67].

Akin to VL, IDDO has also recently engaged with the research community to assess the feasibility of undertaking IPD-MA using PKDL studies to address questions regarding risk factors, drug safety and efficacy [12]. This systematic literature review has provided additional resources to generate a descriptive summary on different epidemiological aspects of PKDL, such as lesion type and lesion healing time [68].

#### Drug safety

The safety of anti-leishmanial drugs has always remained a pertinent cause of concern [69]. The IDDO VL data platform can therefore play a crucial role in knowledge generation and support specific safety issues, such as assessment of ototoxicity (paromomycin) or investigating liver function changes. The IDDO VL platform is currently facilitating an IPD-MA on haematological safety [70].

Of note, ocular safety following PKDL treatment has come to the forefront in recent years, as several recent reports have described eye complications in patients treated with miltefosine in the ISC region [71–77]. A recent observational study of 300 patients with PKDL reported ocular adverse events in 11 (3.7%) patients, of which 3 had persistent partial loss of vision [78]. Incidentally, retinal degeneration in rodents was observed when miltefosine was initially tested for VL treatment [79]. These concerns raised during initial development were overturned following the review of a large series of clinical trials that evaluated a short duration of miltefosine (mostly 28 days or less) in ISC that endorsed its retinal safety [80–87]. However, the recent safety concerns regarding miltefosine are regarding its usage in the management of PKDL, which has a prolonged duration of treatment, and most of the PKDL-associated ocular toxicities were reported after use for longer periods [78]. IDDO is currently systematically reviewing the published literature to create a database of reported ocular adverse events for VL and PKDL (PROSPERO registration: CRD42023463988).

#### Future research

Several other areas were identified for which, at present, there are insufficient data to address the research questions. Extension of the platform to cutaneous leishmaniasis (CL) and incorporation of immunological studies in the data platform remain part of the proposed future research. In particular, extension of the platform to incorporate observational datasets generated from routine practice remains crucial. This is currently being undertaken in the context of Sudan (University of Khartoum’s IEND database) and represents an important initial step towards characterisation of the patient spectrum, which can help generate key insights regarding treatment effectiveness in patient populations frequently excluded from clinical trials. For example, safety concerns have led to systematic exclusion of pregnant and lactating women, individuals with severe anaemia [88] and women of child-bearing age who are unwilling to accept contraception from clinical trials. Patients who present with a relapse are also largely excluded from existing clinical trials. Careful assessment of these valuable datasets generated from observational settings can provide important insights regarding the real-world effectiveness of the drugs in the population excluded from trials. In addition, methodological innovation by incorporating excluded patients in a future pragmatic trial can help in generating evidence in these neglected patient groups.

## Discussion

In 2005, an elimination agenda was set out by India, Bangladesh and Nepal in a landmark cross-border collaboration to eliminate VL by 2015 and reduce the cases to less than 1 in 10,000 at the block level [89]. The ensuing decade saw a successful decline in the annual VL incidence, and efforts are currently underway to consolidate the gains made [90, 91]. However, there are views attributing the current elimination of VL to cyclical trends in the past. For example, VL literature has pointed towards the re-emergence of VL from near eradication in the Indian state of Bihar beginning in the early 1970s and a peak pattern of disease outbreak being observed (peaks observed in the 1930s, 1970s and 1990s) [92]. It has to be pointed out that the remission of the VL endemicity in the 1950s was the collateral benefit of extensive DDT spraying organised by the National Malaria Eradication Programme. There has been near absence of the VL in the state of Assam in India in the current epidemic (the state was severely affected in the past epidemics with thousands of deaths). However, no emergence of any new epidemic in Assam has occurred even after > 50 years of its onset in the state of Bihar, and has been attributed to absence of the vector *Phlebotomus argentipes* [93]. Thus, intense vector control and mapping the vector distribution [67] and diagnosis and treatment of any remaining cases, combined with effective surveillance, remain key to preventing the return of VL epidemics. This is particularly relevant as recent reports have suggested the emergence of new foci in Nepal, despite the number of cases remaining low [94]. Another important consideration is that, as the disease is in the elimination phase, there is a gradual decline in host immunity. The lack of immunity was thought to be an important contributing factor to the epidemic in the western upper Nile [95] and the blue Nile [96] in East Africa. Thus, the recent epidemiological gains in ISC should be constantly monitored owing to the epidemic potential of the disease [97].

A major challenge is the lack of alternatives to existing treatment regimens. Currently, only one novel drug, LXE408, co-developed by Novartis and DND*i*, is in phase II clinical development, with trials ongoing in India and Ethiopia. A few other compounds remain in earlier stages of development [98]. Similar to other NTDs, because of the lack of viable economic prospects, drug development for VL is limited. Furthermore, patient recruitment for clinical trials in the ISC is nearly impossible owing to a steep decline in the incidence of the disease, and remains equally challenged in East Africa owing to political instabilities, such as the ongoing conflicts [99]. In addition, as the global health investment and research and development (R&D) funding is rapidly shrinking, optimising current resources to accelerate innovation and extending the therapeutic shelf-life of the existing drug regimens is extremely important. Maximising existing data to generate new evidence is a practical and cost-effective option and can contribute to accelerating the development of new therapeutic options.

The initial aim of the IDDO VL data platform was to facilitate the sharing, curation, standardisation and archiving of existing clinical data on drugs and treatments for VL. This initial aim has largely been achieved as data from more than 50 VL and PKDL studies are hosted in the platform (nearly 15,000 patients) [24]. The IDDO VL data platform has now reached a critical mass of data to address public health questions of interest, and is currently facilitating IPD meta-analyses on drug safety and efficacy [44, 45, 70]. The current VL data platform was initially developed to capture clinical trial datasets for which individual participant data were available. However, trial data often exclude patients in real-world or programmatic settings who fall outside trial eligibility criteria. In this direction, the IDDO VL data platform has already expanded to include individual participant data from a few unpublished studies and data from observational settings, although broader real-world and programmatic datasets remain under-represented. Continued efforts to incorporate data from these settings will further enhance the platform’s representativeness and its utility for addressing diverse research and policy questions. Given the challenges in retrieving IPD from historical VL studies, the IDDO VL platform can also serve as a neutral venue for archiving research data. In addition, the open-access VL and PKDL clinical trial libraries have attempted to address some of the questions identified in the research agenda through aggregated analysis on different aspects of drug safety and efficacy [21, 31, 69, 88].

## Conclusions

The development of a global research agenda for visceral leishmaniasis has provided an inventory of priority research questions of public health importance to complement the ongoing global disease control efforts. Some of these questions are best addressed through a collaborative individual participant data meta-analysis from clinical trials harmonised to common standards. As new drug compounds progress through the clinical development pipeline and as the search for a vaccine still continues, the secondary re-use of data hosted in the IDDO VL platform can support methodological innovation, serve as a historical control and guide the design of future trials. Sustained collaboration and investment are required to maximise the value of this platform and contribute to the elimination of VL and management of PKDL. The experiences shared in this review can serve as an exemplar for researchers interested in undertaking IPD-MA in the context of other NTDs and for the broader infectious diseases in general.

## Supplementary Information

**Additional File 1.**

## Data Availability

This article describes methodology, and there were no data used. No datasets were generated or analysed during the current study.
