# Supplementary material for: The development of a global research agenda and individual participant data platform for visceral leishmaniasis: challenges and future opportunities
Source: Parasit Vectors. 2025 Dec 2;18:496. doi: 10.1186/s13071-025-07121-2 (PMC12673693; doi:10.1186/s13071-025-07121-2)
Supplement: Supplementary file 1 — Additional File 1. [file 13071_2025_7121_MOESM1_ESM.pdf]

IDDO
INFECTIOUS DISEASES DATA OBSERVATORY

Visceral Leishmaniasis Research Agenda

Version 1.0 – July 2019

OVERVIEW

The proposed Visceral Leishmaniasis (VL) research agenda set out below has been developed by the VL data platform Scientific Advisory Committee (SAC – see Appendix for membership). The purpose of this agenda as it relates to the IDDO VL data platform is outlined as follows:

(i) The IDDO VL data platform is facilitating the sharing, curation, standardisation and archiving of existing clinical data on drugs and treatments for VL, including diagnostic methods.

(ii) This research agenda is designed to guide the utilisation of data shared on the VL data platform and encourage collaboration and involvement of the leishmaniasis scientific community with this resource.

(iii) This agenda should reflect priority research areas signposted by the research community. It aims to identify research questions related to VL that were not/or cannot be satisfactorily addressed in single studies and are considered by the scientific and public health community of urgent concern. IDDO will support the development of individual patient data (IPD) meta-analyses of these archived data for specific priority research questions.

(iv) The targeted data sets are primarily clinical trials (see Bush et al., 2017[i]), but may also include data sets from descriptive clinical studies reporting on patient cohorts of VL. Importantly, the requested and shared data sets are individual patient-level (IPD) data and include but are not limited to demographic, clinical, laboratory, adverse event and treatment related outcome measures.

(v) This agenda will be a working document and as research priorities are addressed and new prospective data collected, additional questions and capabilities for analysis will arise.

(vi) This research agenda will be updated as required, with subsequent development guided by the SAC and wider research community.

(vii) The development of this research agenda will be following a three tier process:

a. First tier: first draft developed by the VL data platform SAC (August – October 2018).
b. Second tier: version sent for comments to a larger audience of identified VL experts (November 2018 – April 2019).
c. Third tier: version posted on the IDDO website for 2 months for additional comments (May – June 2019).

We have completed the first two tiers of the process and the VL community has identified three priority areas for research for which sufficient datasets are available and outcomes should be available

within 2–3 years. Several other areas were also identified for which at present there is insufficient data to formulate the requisite research questions.

PRIORITY AREAS

The priority areas identified to guide the utilisation of data shared on the VL data platform are:

1 Methodological questions

The impact of methodological variations in the derived estimate of anti-leishmanial drug efficacy has received insufficient attention. What is the effect of this methodological variation on the derived estimate of drug efficacy and effectiveness? Attention needs to be on several study design issues, including:

- Study inclusion and exclusion criteria
    - Describe how inclusion and exclusion criteria are representative of affected populations per region
    - What anthropometric indicators are used for patient selection/exclusion in clinical trials (e.g. BMI vs. MUAC)?
- Case ascertainment. Details of the microscopy/laboratory/staining methods used (e.g. source of aspirates spleen/bone marrow; method used for species differentiation etc)
    - Describe the quality control process in laboratory
    - Describe if external quality assurance (EQA) – particularly for microscopy was conducted
    - Evaluation of the performance of serological methods – rapid test compared to standard methods.
- Quality of pharmaceutical product, how is it assured and documented?
- How are the primary and secondary endpoints defined?
- How does the follow-up duration affect derived efficacy? What is the optimal duration of study-follow-up required to capture the treatment failures with different therapies in Asia/Africa?
- What if PKDL is considered as a late outcome, how does this affect the efficacy estimates?
- What are the methods used for sample size/power calculation?
- What statistical method was used for analysing and defining endpoints?
- What are/should be the endpoints. Death/survival, parasitological/spleen size, haematological, inflammation, etc?
- How are analysis populations defined?
- How are subgroups for analyses defined?
- How completed trials reported their process including:
    - Ethics review
    - Regulatory review (incl. GCP, GCLP and local regulatory compliances)
    - Expected risks and potential benefits
    - Informed consent process
    - Confidentiality and privacy
    - Respect for study participants (including how findings are shared with them)
    - Study team composition, training and supervision
    - Study procedures
    - Laboratory sample collection and storage

- Patient costs and reimbursement
- Safety considerations and rescue treatment
- Safety reporting
- Monitoring and Quality Control
- Resources and support
- Trial registration

It is expected that the VL data platform will stimulate methodological advances and foster more standardised approaches.

2 a Clinical features, treatment, and outcomes

- What are the anthropometric/hematological/inflammatory values at time of VL diagnosis? Is there significant variation by region/country?
- Is it possible to characterise the evolution of hematological profiles during the treatment and study follow-up period?
- Can we describe the outcomes of molecular clearance tests from blood or tissue?
- What are the risk factors affecting treatment outcomes:
    - How variations of parasites and human polymorphism variations in different regions impact on clinical study outcomes
    - How do the different VL parameters (inflammation, anthropometric, spleen/liver size, hematological, parasitological) during and after treatment impact upon time to clinical recovery?
- What are the most appropriate outcome measures of improvement for different parameters during treatment (fever, weight, spleen size, haemoglobin, etc.)?
    - Describe evolution of clinical symptoms such as spleen size, fever clearance, asthenia.
- What is the ideal timing and method of the "Test-of-Cure" (TOC), for clinical trials, but also for recommendations in routine clinical settings: When is a TOC indicated? How should this be done?
- What is the definition of VL relapse (vs. definition of initial treatment failure, recrudescence, etc.)?
- What is the temporal incidence of VL relapse? Is it possible to predict a relapse?
- What should be the definition of relapse or reinfection in a case of VL?
- What are biomarkers for prediction of VL relapse (anthropometric, spleen size, haematological, parasitological)?
- What are the effects of different treatment regimens, including combination therapy, on the incidence and timing of relapse?

If there is sufficient information, it would be of importance to provide:

- Description of co-morbidities (such as malaria, tuberculosis, HIV and others) and their association with clinical outcome.
- Description of supportive treatments, e.g. blood transfusion for special cases, antibiotic treatment, nutritional care. What are their impacts?
- Description of concomitant treatments, e.g. antiretroviral, antidiabetic, antituberculosis therapies. What are their impacts?
- Description of outcomes by sub-populations, for example:
    - Description of clinical outcomes in paediatric populations and other age groups
    - Which is the most suitable drug (safe and effective) to be given to a pregnant woman with VL?

2 b PKDL

- What are the thresholds of infectivity for PKDL?
- What are the optimal confirmatory diagnostic signs?
- What is relapse and treatment failure in the case of PKDL?
- What is the optimal duration of treatment for PKDL, for example until the complete clinical disappearance of the lesions or until there is parasitological cure by microscopy in the skin lesions?
- What should be the treatment guideline if a patient stops treatment before completing the full course of the drug (miltefosine for 12 weeks)?
- What treatment should be given after the patient relapses following a full course of miltefosine or AmBisome?
- What should be the treatment guideline for a VL case coexistent with PKDL?
- What are the effects of different treatment regimens on the incidence and timing of PKDL?

3 Safety / pharmacovigilance / deaths

- How is the safety profile of antileishmanials used for clinical studies and in policy?
- How could a review of Adverse Drug Reactions (ADRs) and Severe Adverse Events (SAEs) from National pharmacovigilance (PV) information (Asia) be used or implemented within a PV system?
- What are the systems to detect rare side effects of drugs e.g. US-FDA cure programme?
- Does AmBisome induce hypokalaemia or other cardiotoxic safety concerns?
- What is the feasibility of the development of a death registry?
    - How does it feed the PV WHO database?
- What are risk factors determining deaths?
    - Definition of death due to VL with or without co-morbidity.
- Why is VL developing more severely in different areas in the same country? Is there a relationship between severity of disease and distribution of the parasites?

4 Parasite database

For some of the clinical studies included in the IDDO VL-data platform, the systematic collection of molecular data from parasites taken in clinical isolates will enable further research questions:

- How can this data be used to link clinical to epidemiological studies?
- How can molecular data be integrated into research to identify bio-markers (for example, drug resistance)?
- How can parasite strain tracking be used to support outbreak studies?

FUTURE RESEARCH

A further series of areas for research have been identified, which will be regularly reviewed and added to the research agenda when further data has been gathered that can frame more appropriate questions. These areas are:

- VL in pregnancy
    - Importance of tracking symptomatic cases
    - Early detection of VL in children (vertical transmission)
    - New foci of CL
    - New foci of VL
- VL-HIV co-infections

- Which is the most suitable drug (safe and effective) or combination to be given in a case of HIV-VL co-infection, so that there is effective survival at least for five years, no relapses and no drug interactions?
- VL and immunocompromised patients
  - Impact of any condition leading to immunosuppression on VL treatment outcomes, in particular transplants.
- Pharmacokinetics
  - Description of PK drug profiles available including unpublished data
- Disease progression
- Biomarkers and diagnostics
  - Their use in pharmacodynamics
  - Treatment failure/drug resistance
  - Link with database on parasite genetic diversity (NGS should be considered)
- Test of cure for VL and PKDL
- Infection Risk and epidemiology
  - Outbreak research (particularly relevant in a post-elimination phase)
- Nutritional status
- Vaccine studies in CL/CL
  - Sudan is already conducting vaccine studies for PKDL
  - In future we may have vaccine studies for VL and CL
- Studies documenting parasite presence on the skin
  - Thought that VL parasites are present on the Langerhans cells on the skin
- Social Science
  - Social science dimension of VL
  - Improvement in cognitive skills for children after VL treatment?
- Blood transfusion and VL
- VL and TB coinfection
  - Which is the most suitable drug (safe and effective) or combination to be given in a case of HIV-TB co-infection?
- Asymptomatic VL

APPENDIX 1 SAC MEMBERSHIP

Scientific Advisory Committee

Ahmed Musa; Carlos Costa; Dinesh Mondal; Jorge Alvar; Koert Ritmeijer; Marleen Boelaert; Shyam Sundar; Simon Croft (Chair)

Secretariat

Philippe Guerin, Brittany Maguire, Michael Otieno, Sauman Singh, Matthew Brack

[i] Bush JT, Wasunna M, Alves F, Alvar J, Olliaro PL, Otieno M, et al. (2017) Systematic review of clinical trials assessing the therapeutic efficacy of visceral leishmaniasis treatments: A first step to assess the feasibility of establishing an individual patient data sharing platform. PLoS Negl Trop Dis 11(9): e0005781. https://doi.org/10.1371/journal.pntd.0005781
